# Catalogue and Circular of the Medical Department of Laporte University

**Published:** 1842-12

**Authors:** 


Art. VI.—*Catalogue and Circular of the Medical Department of Laporte University.* First Session, 1842. Laporte, T. A. Stewart.

We were not a little surprised to find on our table, some weeks ago, a small document bearing the above title. Of a truth, we would as soon have thought of looking for Pompey's pillar as for a medical school at Laporte, Indiana, twelve miles from Lake Michigan. Ten years ago, the place where the town now stands, and the whole region round-about, was an immense wilderness, whose silence was unbroken save by the screech of an owl, or the yell of a Pottawatomie. Within these short ten years—nay, in a much shorter period —the country has been reclaimed from a state of nature: lands have been surveyed, and marked off into counties; towns have been built; farms improved—every thing, in short, has been added and done, that a dense and busy population, collected silently but swiftly from all quarters of the Union, could add or could do. And here last of all comes a medical school, which, like our Louisville Medical Institute, is designed to be the nucleus of a large and flourishing University.

Many perhaps who read this, and who, like ourselves, have been negligent of the rapid progress of improvement in the whole north-west, will smile as we did on first seeing this announcement. But let none such be deceived. If they will take a map of the United States, they will perceive that Laporte University stands in the centre of an immense region of country: Northern Indiana, the northern part of Ohio and Illinois, the whole of Michigan, Iowa, Wisconsin—a vast region indeed, already occupied by a large population, and capable of sustaining a much larger one, which must be supplied with physicians and surgeons. The enterprise, then, evinces more of judgment and prevision, than might at first be supposed. Moreover, the spirit and energy that have thus far characterized the founders of this university, are not merely deserving of all praise, but give favorable augury for the future. The charter was procured about the middle of last winter; operations were immediately commenced, and *courses of lectures delivered to a medical class of fifteen pupils, and a law class of ten;* and since that time, as we are informed, a commodious Medical Hall has been erected, which is doubtless now occupied.

The Medical Department is organized as follows:

Daniel Meeker, M. D., *Professor of Anatomy, Physiology and Surgery, and Dean of the Faculty;* G. A. Rose, M. D., *Professor of the Theory and Practice of Medicine:* J. P. Andrews, M. D., *Professor of Obstetrics and the Diseases of Women and Children;* Franklin Hunt, M. D., *Professor of Materia Medica, Botany and Medical Jurisprudence;* J. B. Niles, A. M., *Professor of Chemistry and Natural Philosophy;* J. G. Newhouse, M. D., *Demonstrator of Anatomy.* The requirements for graduation are similar to those of the best regulated schools in the country. The candidate must be twenty-one years of age, of good moral character, have studied medicine three years, including two full courses of lectures, one of which must have been in this institution. Three years' practice and one course of lectures will entitle the student to an examination for a degree.

Attached to the circular are some complimentary resolutions passed by the class at the close of the session. We copy the last in the series, as being at once novel and characteristic:

"Resolved—that we now adjourn to meet again in this place on the second Monday of next November."

And we have no doubt that every mother's son of them have done it! Your Hoosier is not easily baffled; we speak from a twenty years' knowledge of the people. C.

